# Integrated Transcriptomic and Metabolomic Analyses Reveal Leaf Regulatory Mechanisms Underlying Low-Nitrogen Tolerance in Oat (*Avena sativa* L.)

**DOI:** 10.3390/cells15201874

**Published:** 2026-10-15

**Authors:** Jing Pan, Miaomiao Huang, Zeliang Ju, Xiang Ma, Lianxue Duan, Long Ren, Zhifeng Jia

**Affiliations:** 1 Laboratory for Research and Utilization of Qinghai Tibet Plateau Germplasm Resources, Academy of Agriculture and Forestry Sciences, Qinghai University, Xining 810016, China; panjingoffice@163.com (J.P.);; 2 Key Laboratory of Superior Forage Germplasm in the Qinghai-Tibet Plateau, Qinghai Academy of Animal Husbandry and Veterinary Sciences, Qinghai University, Xining 810016, China; 3 School of Computer Technology and Application, Qinghai University, Xining 810016, China

**Keywords:** *Avena sativa*, low-nitrogen stress, transcriptomics, metabolomics, RNA-seq

## Abstract

**Highlights:**

**What are the main findings?**

- Low-nitrogen stress caused more pronounced changes in growth, nitrogen accumulation, and transcriptomic and metabolic profiles in the sensitive germplasm ZNY256 than in the tolerant germplasm Jiayan No.2.
- Integrated transcriptomic and metabolomic analyses highlighted nitrogen metabolism, phenylpropanoid and flavonoid biosynthesis, glutathione metabolism, and antioxidant-related processes as major components of the low-nitrogen response.

**What are the implications of the main findings?**

- Jiayan No.2 maintained better growth and nitrogen accumulation more effectively than ZNY256 under low-nitrogen conditions, supporting its value as a germplasm resource for improving nitrogen-use efficiency in oat.
- The candidate genes and pathways identified here provide a basis for further functional studies and for screening nitrogen-efficient oat germplasm.

**Abstract:**

Nitrogen deficiency limits oat growth and productivity, but the molecular basis of contrasting low-nitrogen responses among oat germplasms remains poorly understood. This study compared the low-nitrogen-tolerant germplasm Jiayan No.2 with the low-nitrogen-sensitive germplasm ZNY256 after 15 days of hydroponic cultivation under normal nitrogen (10 mM NH_4_NO_3_) or low nitrogen (1.25 mM NH_4_NO_3_). Growth traits, plant nitrogen content, nitrogen-metabolism-related enzyme activities, soluble sugar content, transcriptomes, and untargeted metabolomes were analyzed. Low nitrogen reduced plant height and plant nitrogen content in both germplasms, with more pronounced changes in ZNY256. Root length was unaffected, whereas root dry weight decreased only in ZNY256. ZNY256 also showed a larger transcriptional response than Jiayan No.2. Functional analyses highlighted phenylpropanoid and flavonoid biosynthesis, glutathione and nitrogen metabolism, ribosome biogenesis, and plant MAPK signaling. Metabolomic profiling identified changes in amino acid-, lipid-, secondary metabolite-, and antioxidant-related features. Integrated analysis and qRT-PCR validation further identified candidate genes associated with these responses. These findings characterize transcriptional and metabolic changes associated with the contrasting responses of the two oat germplasms to nitrogen deficiency and provide candidate genes, metabolites, and pathways for further investigation of low-nitrogen adaptation in oat.

## 1. Introduction

Nitrogen is essential for plant growth and agricultural productivity [1]. Nitrogen fertilization can increase crop yield, but low recovery efficiency increases production costs and environmental losses [2,3,4]. Nitrogen fertilizer production is energy intensive, while unassimilated nitrogen may be lost through leaching or gaseous emissions, including nitrous oxide [4]. Improving nitrogen-use efficiency is therefore important for maintaining crop production while reducing fertilizer inputs and their environmental effects. This objective is particularly relevant to forage crops, for which the identification and use of low-nitrogen-tolerant germplasm could support more sustainable production systems [5,6].

Oat (*Avena sativa* L.) is cultivated for both grain and forage, and its grain is valued for its dietary fibre and phytochemical composition [7,8]. Nitrogen limitation can constrain oat growth through changes in biomass production and leaf physiology. In a comparison of five oat germplasms, low nitrogen reduced plant height, shoot dry weight and plant nitrogen content, accompanied by decreases in net photosynthetic rate and nitrate reductase activity [8]. Root responses can differ from those of the shoot. In naked oat (*Avena nuda* L. cv. Baiyan 2), low nitrogen reduced shoot growth, leaf area and photosynthetic performance but increased total root length, root dry weight and the root-to-shoot ratio [9]. These observations indicate that nitrogen limitation can alter biomass allocation rather than uniformly suppress the growth of all organs. Oat germplasms also differ in their physiological responses to nitrogen limitation; a previous assessment ranked Jiayan No.2 as more tolerant than ZNY256 [8]. Such variation provides a basis for examining the transcriptional and metabolic responses associated with low-nitrogen tolerance in oat leaves.

Nitrogen limitation restricts plant growth by altering root development, photosynthesis, and carbon and nitrogen metabolism [10]. Root architectural responses may include changes in primary root elongation, root diameter, and root density [11]. Nitrogen deficiency can also reduce amino acid, soluble protein, and proline accumulation [12] and impair chlorophyll formation and photosynthetic performance [13,14,15]. Previous studies of low-nitrogen tolerance in oat have focused mainly on seedling phenotypes, physiological traits, yield, quality, and nitrogen-use efficiency, whereas the associated molecular and metabolic responses remain less well characterized [16]. Here, two oat germplasms with contrasting low-nitrogen responses were examined using physiological measurements, RNA sequencing, and untargeted metabolomics. The study aimed to identify transcriptional and metabolic features associated with their contrasting responses to nitrogen limitation.

## 2. Materials and Methods

### 2.1. Plant Materials and Low-Nitrogen Treatment

The low-nitrogen-tolerant oat germplasm “Jiayan No.2” and the low-nitrogen-sensitive germplasm “ZNY256” were selected from oat seedling resources evaluated for low-nitrogen tolerance. Both germplasms were maintained at the Academy of Animal Science and Veterinary, Qinghai University. Seeds of similar size were selected and grown hydroponically. Normal-nitrogen and low-nitrogen treatments contained 10 and 1.25 mM NH_4_NO_3_, respectively. Plants were exposed to the treatments for 15 days before physiological measurements and leaf sampling. Each germplasm × nitrogen treatment combination consisted of three independent hydroponic containers, with 10 plants grown in each container. Each container contained 5 L of nutrient solution and was regarded as one independent biological replicate (*n* = 3); individual plants within the same container were therefore not treated as independent replicates. A modified nitrogen-free Hoagland nutrient solution (HB8870-9; Qingdao Hope Bio-Technology Co., Ltd., Qingdao, China) was used as the basal solution. According to the manufacturer, the basal medium contained 152.828 mg L^−1^ KCl, 456.638 mg L^−1^ K_2_HPO_4_·3H_2_O, 246.48 mg L^−1^ MgSO_4_·7H_2_O, 11.17 mg L^−1^ NaFe-EDTA, 1.546 mg L^−1^ H3BO3, 0.338 mg L^−1^ MnSO_4_·H_2_O, 0.125 mg L^−1^ CuSO_4_, 0.576 mg L^−1^ ZnSO_4_·H_2_O, and 0.102 mg L^−1^ Na_2_MoO_4_. NH_4_NO_3_ was added to the basal solution to obtain normal-nitrogen (CK, 10 mM NH_4_NO_3_) and low-nitrogen (LN, 1.25 mM NH_4_NO_3_) treatments. The nutrient solution was adjusted to pH 5.8 and renewed every 3 days. Plants were grown under a 16 h light/8 h dark photoperiod at 25/20 °C (day/night). After 15 days of treatment, the second fully expanded leaves from five plants within each hydroponic container were pooled to form one biological sample. Thus, three independent pooled samples were obtained for each treatment combination and used for transcriptomic and metabolomic analyses.

After 15 days of treatment, the second fully expanded leaf from the top of each plant was collected. Four groups were analyzed: ZNY256_CK, ZNY256_LN, Jiayan No.2_CK, and Jiayan No.2_LN. Each group comprised three independent biological replicates, giving 12 samples in total. Plant nitrogen content was determined using the Kjeldahl method. Glutamine synthetase (GS), nitrate reductase (NR), starch phosphorylase (SP), and soluble sugar levels were determined using commercial assay kits (Suzhou Comin Biotechnology Co., Ltd., Suzhou, China) according to the manufacturer’s instructions. Samples were immediately frozen in liquid nitrogen and stored at −80 °C until RNA and metabolite extraction.

### 2.2. RNA-Seq and Data Analysis

Total RNA was extracted from oat leaves using TRIzol reagent (Invitrogen, Carlsbad, CA, USA) according to the manufacturer’s instructions. RNA quality and integrity were assessed by 1.2% agarose gel electrophoresis and an Agilent 2100 Bioanalyzer (Agilent, Santa Clara, CA, USA), and RNA concentration was measured using a NanoDrop spectrophotometer (Thermo Fisher Scientific, Waltham, MA, USA). Samples with concentrations > 400 ng μL^−1^, OD260/280 ratios of 1.8–2.1, and RNA integrity numbers > 7 were used for library preparation. Transcriptome sequencing was performed by Novogene Co., Ltd., Beijing, China.

Raw sequencing reads were quality-filtered and pre-processed using fastp (v1.4.0), and the quality of the resulting clean reads was assessed using FastQC (v0.11.9). Clean reads were aligned to the hexaploid oat (*Avena sativa* L.) reference genome Oat_OT3098_v2 (INSDC assembly accession GCA_022788535.1) using HISAT2 (version 2.1.0) with default parameters. The reference genome and gene annotation were obtained from Ensembl Plants release 63 (June 2026); the OT3098 annotation was imported from the external annotation generated for this assembly (Ensembl database version 116.1; data source: PepsiCo). Gene-level raw read counts were generated using featureCounts (v2.0.3). Differential-expression analysis was performed in DESeq2 using the raw gene-level count matrix as input; count normalization and statistical modeling were performed internally by DESeq2. Genes with |log2 fold change| ≥ 1 and a false discovery rate (FDR) < 0.05 were considered differentially expressed. FPKM (fragments per kilobase of transcript per million mapped fragments) values were calculated separately to describe relative transcript abundance and were used only for expression visualization and descriptive analyses, rather than as input for DESeq2 differential-expression testing. GO and KEGG pathway enrichment analyses were subsequently performed to characterize the biological functions and pathways represented by the DEGs.

### 2.3. Untargeted Metabolomics and Data Analysis

Untargeted metabolomic profiling was performed by Novogene Co., Ltd. using LC–MS/MS. The experimental procedures described below are based on the provider’s project report and accompanying workflow documentation. Untargeted metabolomic analysis was performed using liquid chromatography-tandem mass spectrometry (LC-MS/MS). The analysis used the same 12 leaf samples as the transcriptomic analysis: ZNY256_CK, ZNY256_LN, Jiayan No. 2_CK, and Jiayan No. 2_LN, with three independent biological replicates per group. A quality-control (QC) sample was prepared by mixing equal volumes of all experimental samples. The QC sample was injected before, during, and after the analytical sequence to monitor instrument stability and analytical repeatability.

Differentially expressed genes (DEGs) and differentially accumulated metabolites (DAMs) were identified independently using the predefined statistical thresholds. DEGs and DAMs were annotated against the KEGG database and mapped to KEGG pathways. Pathways containing both DEGs and DAMs were identified as transcriptome–metabolome overlapping pathways. The corresponding genes and metabolites were jointly visualized to summarize their pathway-level distribution. This analysis was used to identify biological processes represented in both datasets.

Raw mass-spectrometry data were processed using Compound Discoverer 3.3. Feature detection and cross-sample alignment were based on retention time, mass-to-charge ratio (*m*/*z*), and adduct information. Metabolites were annotated by matching high-resolution MS/MS spectra against the mzCloud and mzVault databases and by comparing full-scan MS information with the MassList database. Metabolite abundance was estimated from chromatographic peak areas and normalized to the total peak area. Features with a coefficient of variation ≥ 30% in the QC samples were excluded. After quality control, 835 positive-ion and 460 negative-ion features were retained. Principal component analysis (PCA) and partial least-squares discriminant analysis (PLS-DA) were used to evaluate overall metabolic variation and group separation. Differential metabolites were selected using variable importance in projection (VIP) > 1.0, fold change (FC) > 1.2 or <0.833, and *p* < 0.05. Metabolites were annotated using the KEGG, HMDB, and LIPID MAPS databases. KEGG pathway enrichment was evaluated using a hypergeometric test, and pathways with *p* < 0.05 were considered significantly enriched. DEGs were defined using |log2FC| ≥ 1 and FDR < 0.05. DAMs were selected using VIP > 1.0, FC > 1.2 or <0.833, and *p* < 0.05, as described above.

To identify metabolite variables contributing to the PCA separation, loading analysis was performed separately on the positive- and negative-ion quantitative matrices from the same 12 leaf samples. For this reanalysis, peak areas were normalized to a total peak area of 1,000,000 for each sample, log2-transformed and Pareto-scaled before PCA. The PCA was restricted to the 12 biological leaf samples, and QC injections were not treated as biological observations. Features were ranked according to the absolute PC1 or PC2 loading, and the 20 highest-ranking features for each component were retained for interpretation.

### 2.4. Weighted Gene Co-Expression Network Analysis

Weighted gene co-expression network analysis (WGCNA) was used to identify groups of genes with similar expression patterns across the oat samples. Genes within selected modules were ranked according to network connectivity, and Cytoscape (v3.9.1) was used to visualize the resulting networks and the 30 highest-ranked candidate genes.

### 2.5. Quantitative Real-Time PCR Validation

Total RNA was extracted using the TaKaRa MiniBEST Plant RNA Extraction Kit (Takara, Kusatsu, Japan). cDNA was synthesized using the Advanced RT-for-PCR Kit (Takara, Japan) and diluted fourfold before amplification. Quantitative real-time PCR was performed using a Bio-Rad CFX Connect Real-Time PCR Detection System (Bio-Rad, Hercules, CA, USA). Each reaction contained 10.0 μL SYBR Green qPCR mix, 0.4 μL each of the forward and reverse primers, and 1.0 μL diluted cDNA. The reaction volume was adjusted to 20 μL with 8.2 μL double-distilled water. Relative gene expression levels were calculated using the 2^−^^∆∆^^Ct^ method, with *GAPDH* as the internal reference gene. Three independent biological replicates were analyzed per group, with three technical replicates for each biological sample. All primers were designed using Primer 5.0, and their sequences are provided in Table S1.

### 2.6. Data Analysis

Statistical analyses of growth and physiological variables were performed using GraphPad Prism (v11.0.1). Data are presented as mean ± SD from three independent biological replicates (*n* = 3), with each hydroponic container representing one biological replicate. The effects of germplasm, nitrogen treatment, and their interaction were evaluated using two-way analysis of variance (ANOVA). A significance threshold of *p* < 0.05 was used.

## 3. Results

### 3.1. Low-Nitrogen Stress Differentially Regulates Growth, Nitrogen Accumulation and Nitrogen Metabolism-Related Enzyme Activities in Oat Germplasms

**Figure 1:**
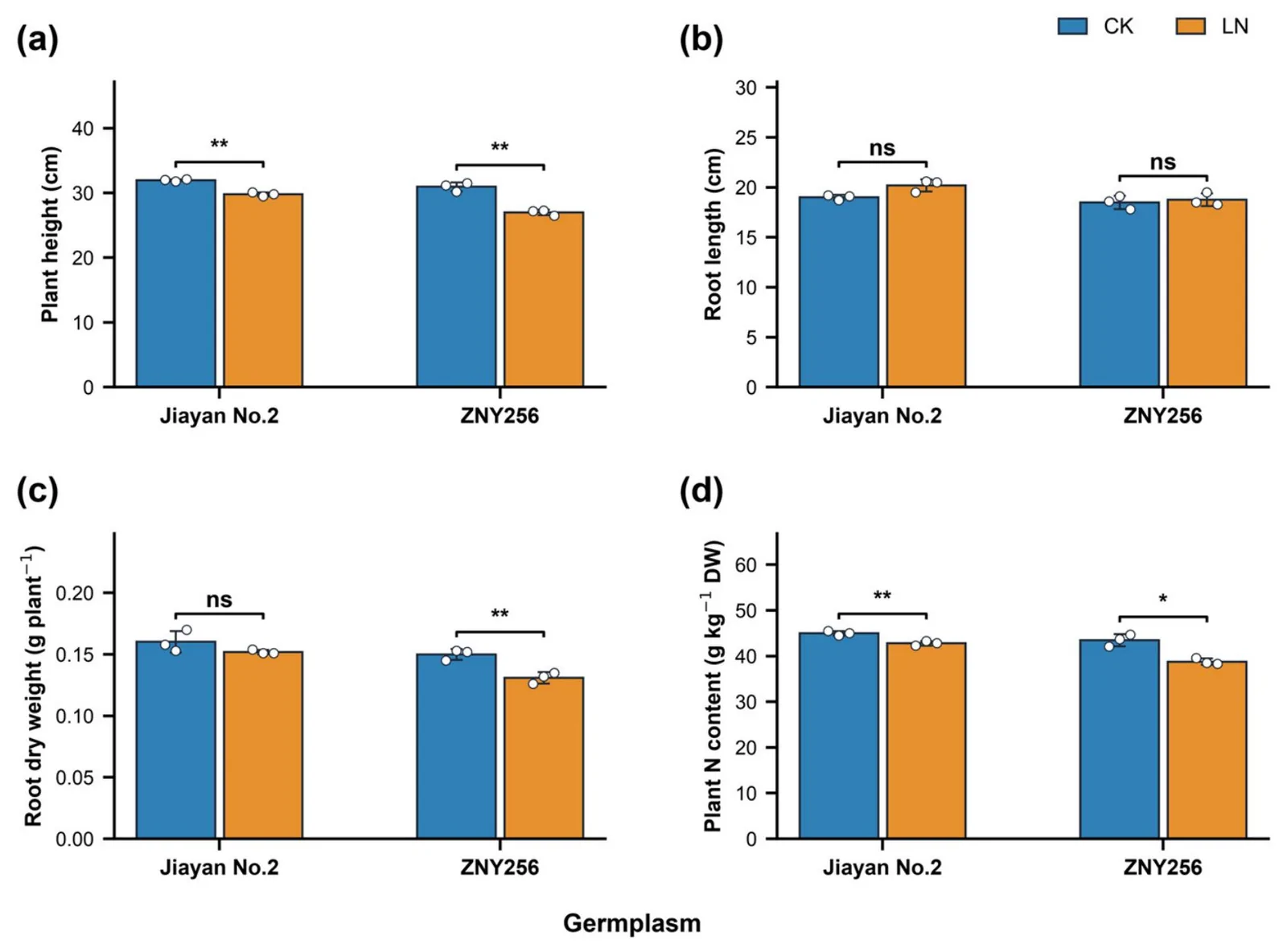
Effects of low-nitrogen treatment on growth and nitrogen accumulation in two oat germplasms. (**a**) Plant height, (**b**) root length, (**c**) root dry weight, and (**d**) plant nitrogen content of Jiayan No. 2 and ZNY256 after 15 days of treatment under normal nitrogen (CK, 10 mM NH_4_NO_3_) and low nitrogen (LN, 1.25 mM NH_4_NO_3_) conditions. Bars represent means ± standard deviation (*n* = 3), with individual biological replicates shown as open circles. Asterisks indicate significant differences between CK and LN within each germplasm (* *p* < 0.05; ** *p* < 0.01; ns, not significant).

After 15 days, low-nitrogen treatment significantly affected the growth of both oat germplasms (Figure 1). Plant height decreased significantly in Jiayan No. 2 and ZNY256, with a more pronounced reduction in ZNY256 (Figure 1a). Root length was not significantly affected in either germplasm (Figure 1b). Root dry weight remained unchanged in Jiayan No. 2 but decreased significantly in ZNY256 (Figure 1c). Plant nitrogen content decreased significantly in both germplasms, and the reduction was more pronounced in ZNY256 (Figure 1d). These responses show that Jiayan No. 2 maintained growth and nitrogen accumulation more effectively than ZNY256 under the low-nitrogen treatment.

**Figure 2:**
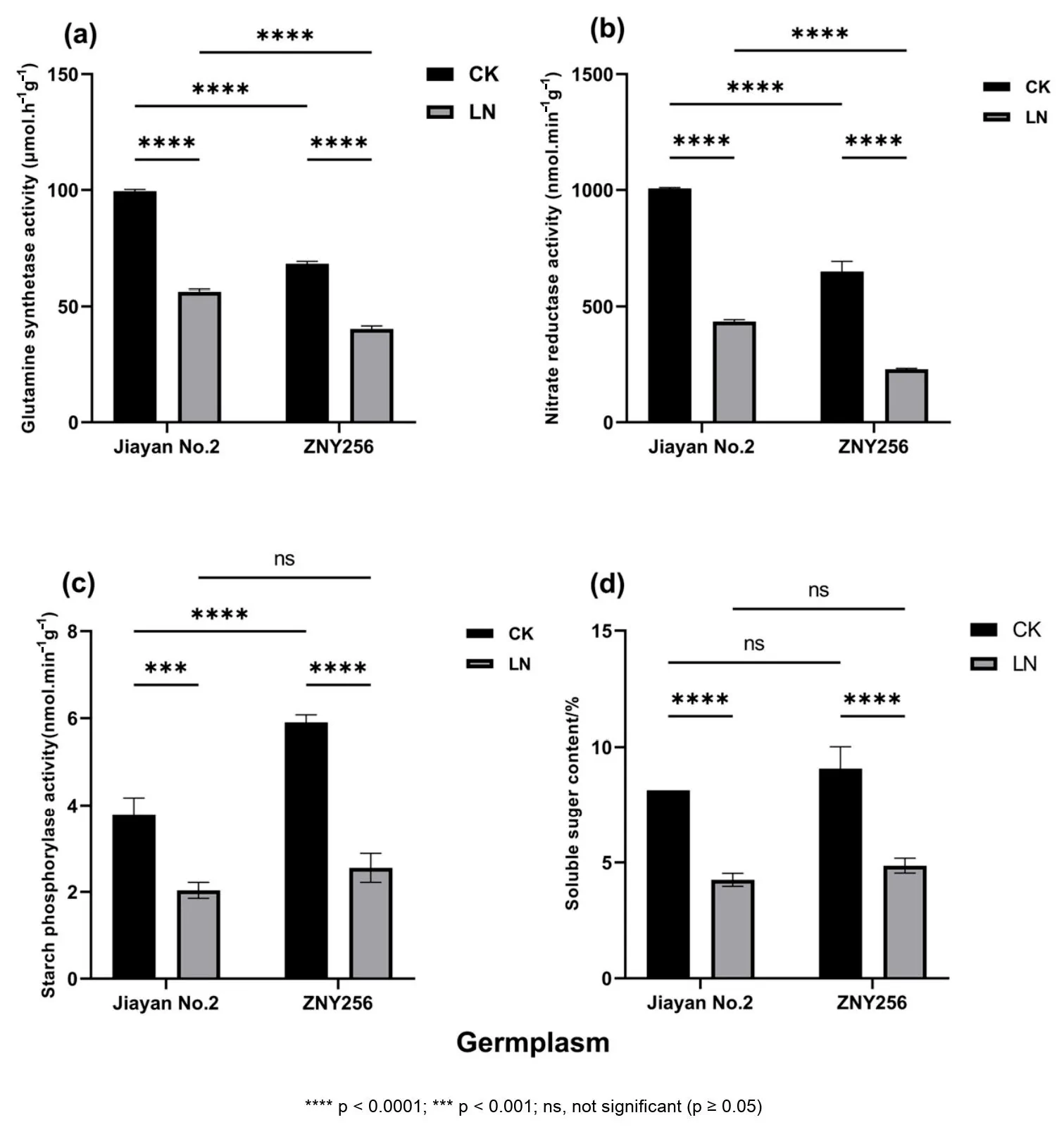
Physiological responses of Jiayan No.2 and ZNY256 under low nitrogen stress. (**a**) Glutamine synthetase activity. (**b**) Nitrate reductase activity. (**c**) Amylopectin phosphorylase activity. (**d**) Soluble sugar content. Data are presented as the mean ± SE (*n* = 3).

At the 15-day endpoint, low-nitrogen treatment altered nitrogen-metabolism-related enzyme activities and soluble sugar content in oat leaves (Figure 2). Glutamine synthetase activity differed between the two germplasms and was lower under low-nitrogen conditions than under normal-nitrogen conditions. Nitrate reductase activity, starch phosphorylase activity and soluble sugar content also showed treatment-associated changes, although the magnitude of these changes differed between germplasms. These endpoint measurements indicate that nitrogen limitation affected both nitrogen assimilation and carbon metabolism in oat leaves. Overall, Jiayan No. 2 showed better maintenance of growth and nitrogen accumulation than ZNY256 under the present low-nitrogen treatment, demonstrating contrasting phenotypic responses of the two germplasms under these experimental conditions.

### 3.2. Transcriptome Analysis Identified Low-Nitrogen Stress-Regulated DEGs

**Figure 3:**
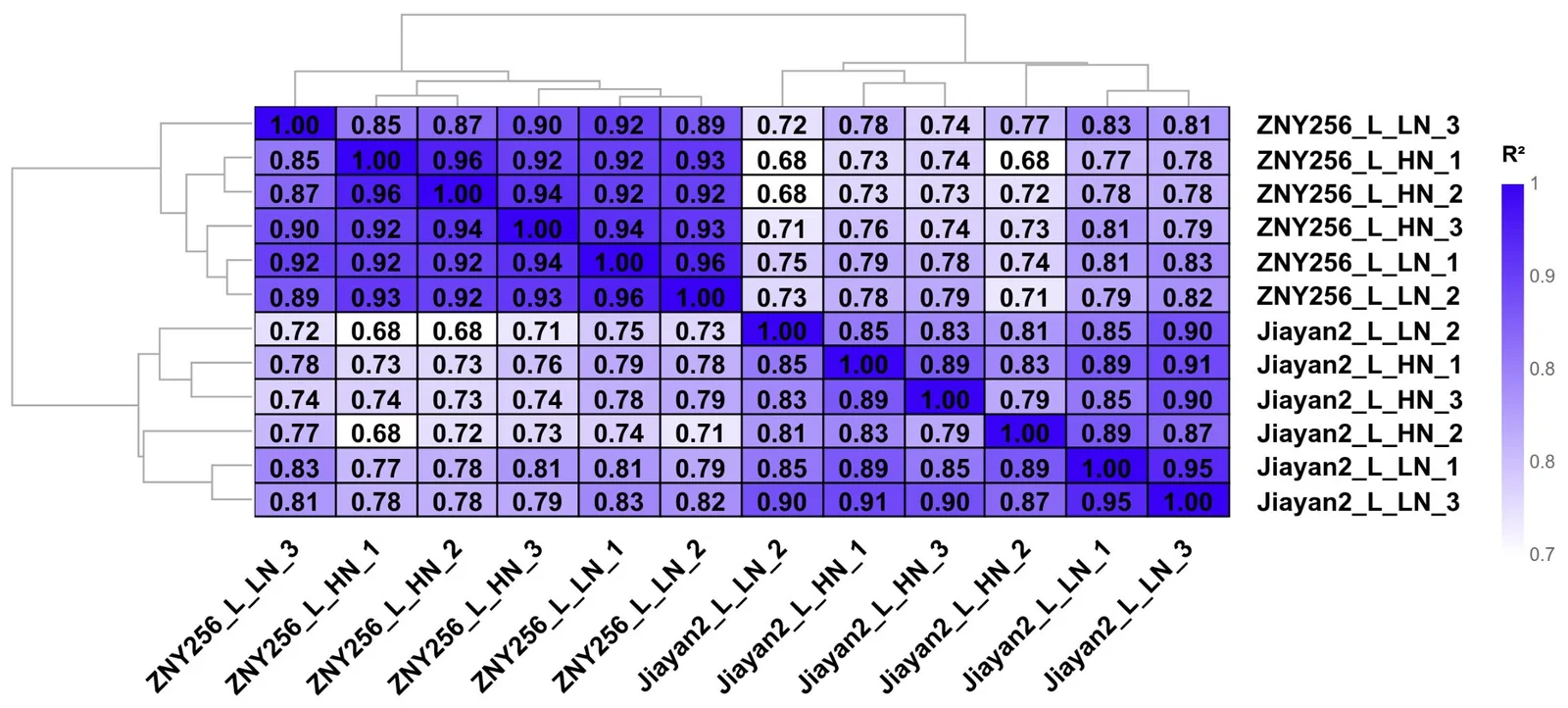
The correlation between different samples. The larger the R^2^ value is, the better the correlation between the samples will be.

Transcriptome sequencing of the 12 oat leaf samples generated 523,937,904 raw reads (78.58 Gb). After quality filtering, 508,489,332 clean reads (76.27 Gb) were retained. Individual samples contained 38.41–46.67 million clean reads and 5.76–7.00 Gb of clean bases, with read-retention rates of 96.26–98.15%. Q30 values ranged from 93.17% to 94.18%, and GC content ranged from 51.87% to 54.54% (Table 1). Pairwise R^2^ values among biological replicates ranged from 0.79 to 0.96 (Figure 3). Hierarchical clustering separated the samples primarily by germplasm, whereas the normal- and low-nitrogen groups partly overlapped within each germplasm. These metrics supported the suitability of the data for downstream analysis while indicating biological variation among some replicates.

Differential expression analysis was performed for four pairwise comparisons (Table 2). Relative to the normal-nitrogen treatment, 1050 genes were upregulated and 2129 were downregulated in Jiayan No. 2 under low nitrogen, yielding 3179 differentially expressed genes (DEGs). In ZNY256, 2438 genes were upregulated and 1922 were downregulated, yielding 4360 DEGs. Thus, ZNY256 showed a larger transcriptional response to low-nitrogen treatment than Jiayan No. 2. Comparison of the two germplasms under low nitrogen identified 6049 genes with higher expression and 8510 genes with lower expression in ZNY256 relative to Jiayan No. 2. Under normal nitrogen, 7204 genes showed higher expression and 10,919 showed lower expression in ZNY256 relative to Jiayan No. 2.

**Figure 4:**
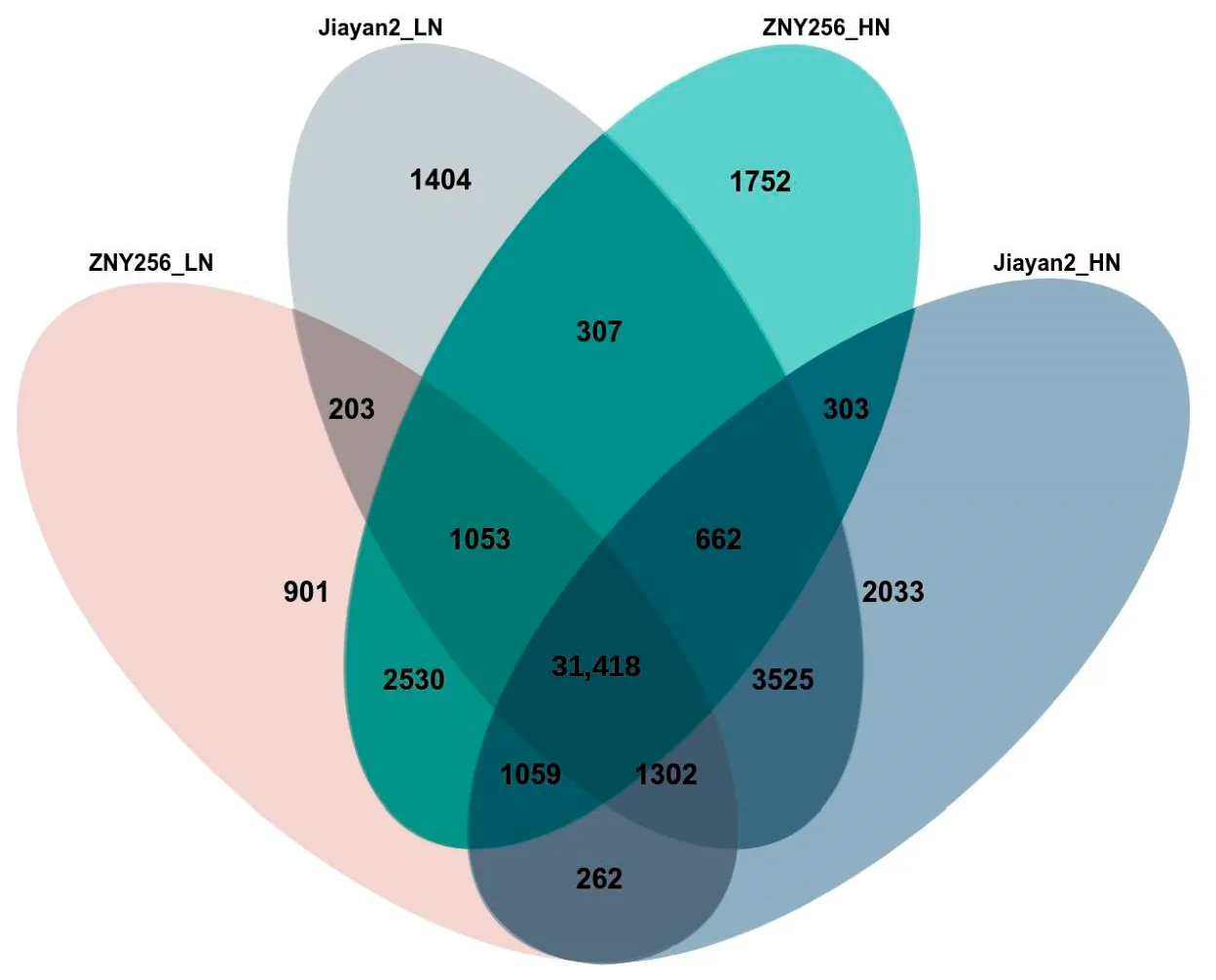
Venn diagram showing genes detected in the ZNY256_CK, ZNY256_LN, Jiayan No. 2_CK, and Jiayan No. 2_LN groups.

Venn analysis was used to compare the sets of expressed genes among the four germplasm-treatment groups. A total of 31,418 genes were expressed in all four groups, representing the core set of genes detected across germplasms and nitrogen treatments (Figure 4). In addition, 901, 1404, 1752, and 2033 genes were uniquely detected in ZNY256_LN, Jiayan2_LN, ZNY256_CK, and Jiayan2_CK, respectively. These group-specific expression patterns further indicated that both germplasm background and nitrogen availability contributed to variation in the leaf transcriptome.

### 3.3. GO Enrichment Analysis of Differentially Expressed Genes

GO enrichment analysis revealed distinct low-nitrogen-responsive functional profiles in the two germplasms (Figure S1). In ZNY256, prominent terms included chitin binding, peroxidase activity, pectinesterase activity, amino sugar catabolism, glucan metabolism, and cell-wall-related processes (Figure S1A). In Jiayan No.2, the highest-ranked terms included calcium ion binding, lyase activity, thylakoid and membrane-related components, aromatic amino acid family metabolism, cell-wall organization, and regulation of hormone levels (Figure S1B). These results indicate that the two germplasms differed in the biological processes and molecular functions represented among their DEGs.

### 3.4. KEGG Enrichment of Differentially Expressed Genes

KEGG enrichment analysis identified six significantly enriched pathways in ZNY256 under low nitrogen (Figure S2A; *p*adj < 0.05): ribosome biogenesis in eukaryotes (ath03008; 41 DEGs), phenylpropanoid biosynthesis (ath00940; 41 DEGs), ABC transporters (ath02010; 26 DEGs), flavonoid biosynthesis (ath00941; 16 DEGs), flavone and flavonol biosynthesis (ath00944; 8 DEGs), and plant MAPK signaling (ath04016; 35 DEGs). In Jiayan No. 2, no pathway reached *p*adj < 0.05 (Figure S2B). Carbon fixation in photosynthetic organisms, glyoxylate and dicarboxylate metabolism, glutathione metabolism, and photosynthesis were therefore interpreted as the highest-ranked pathways rather than as statistically significant enrichment after multiple-testing correction.

### 3.5. Weighted Gene Co-Expression Network Analysis Identifies Candidate Hub Genes Associated with Low-Nitrogen Responses

**Figure 5:**
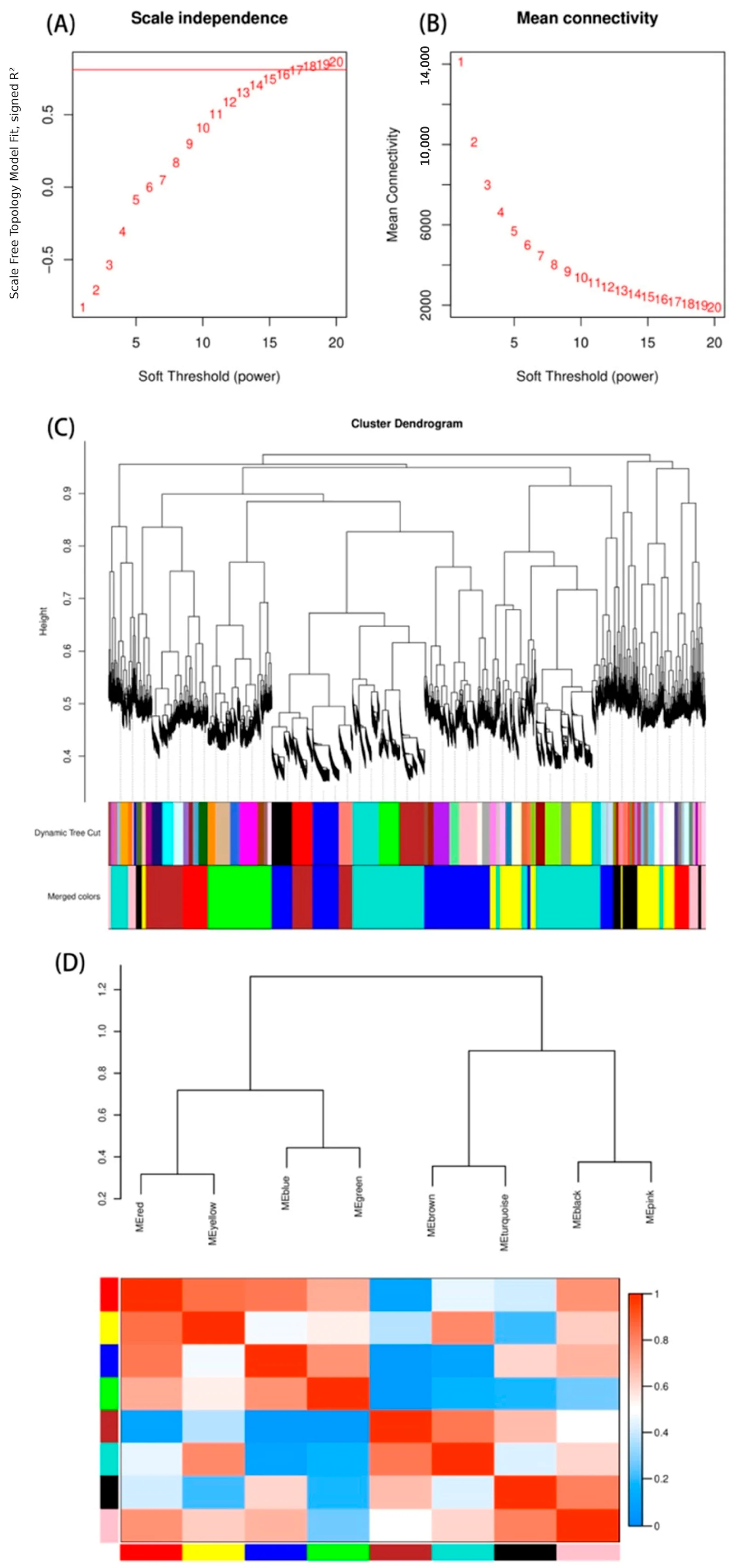
Exploratory weighted gene co-expression network analysis (WGCNA) based on 12 RNA-seq samples. (**A**) The independence of the data scale. (**B**) The average connectivity of the data. (**C**) The hierarchical cluster tree showed 8 modules of co-expressed genes in oat under low nitrogen stress. (**D**) Protein–protein interaction network of co-expressed genes from the turquoise module. In panels (**A**,**B**), the red numbers indicate the candidate soft-thresholding powers, and the red horizontal line in panel (**A**) represents the scale-free topology fit threshold (R^2^ = 0.80). In panel (**C**), different colors represent different gene co-expression modules identified by dynamic tree cutting and subsequent module merging. In panel (**D**), the heatmap colors indicate the degree of correlation between module eigengenes, ranging from blue (low correlation) to red (high correlation).

WGCNA was performed using 25,001 expressed genes (FPKM > 1) to identify co-expression patterns associated with nitrogen treatment and germplasm background. Network topology was evaluated across a range of soft-thresholding powers. A power of β = 18 was selected because it produced an approximate scale-free topology fit of R^2^ = 0.90 while retaining moderate mean connectivity (Figure 5A,B). Hierarchical clustering of the gene-expression profiles resolved eight co-expression modules (Figure 5C). The module dendrogram and eigengene profiles showed distinct expression patterns among the four germplasm–nitrogen combinations, indicating that the modules captured treatment- and genotype-related variation in the leaf transcriptome.

Module–trait relationships were evaluated by calculating Pearson correlations between module eigengenes and the measured traits (Figure 5D). Several modules displayed positive correlations with one another, whereas other module pairs were negatively correlated. These relationships describe coordinated or opposing expression patterns among gene sets; assignment of specific biological functions requires the corresponding module-level enrichment results and trait correlations.

### 3.6. Metabolite Classification and PCA of Oat Leaves Under Low Nitrogen Stress

**Figure 6:**
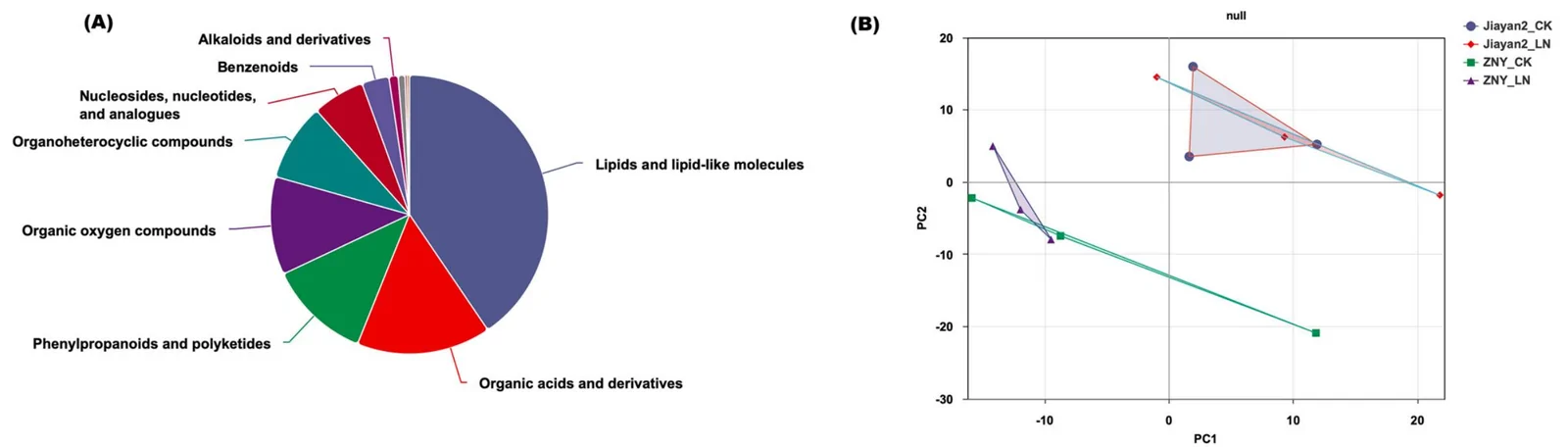
Overview of the oat leaf metabolomic dataset. (**A**) Classification of annotated metabolite features and (**B**) principal component analysis of the 12 samples.

LC-MS/MS analysis retained 1295 metabolite features, comprising 835 positive-ion and 460 negative-ion features. Annotated features represented several chemical classes, including alkaloids, nucleotides, organic heterocyclic compounds, organic oxygen compounds, phenylpropanoids, organic acids, and lipids (Figure 6A). Lipid- and organic-acid-related features accounted for relatively large proportions of the annotated dataset. PCA showed separation trends among the four germplasm-treatment groups in the space defined by the first two principal components (Figure 6B), indicating that both germplasm background and nitrogen treatment contributed to variation in the oat leaf metabolome.

### 3.7. KEGG Pathway Annotation of Oat Leaf Metabolites Under Low Nitrogen Stress

**Figure 7:**
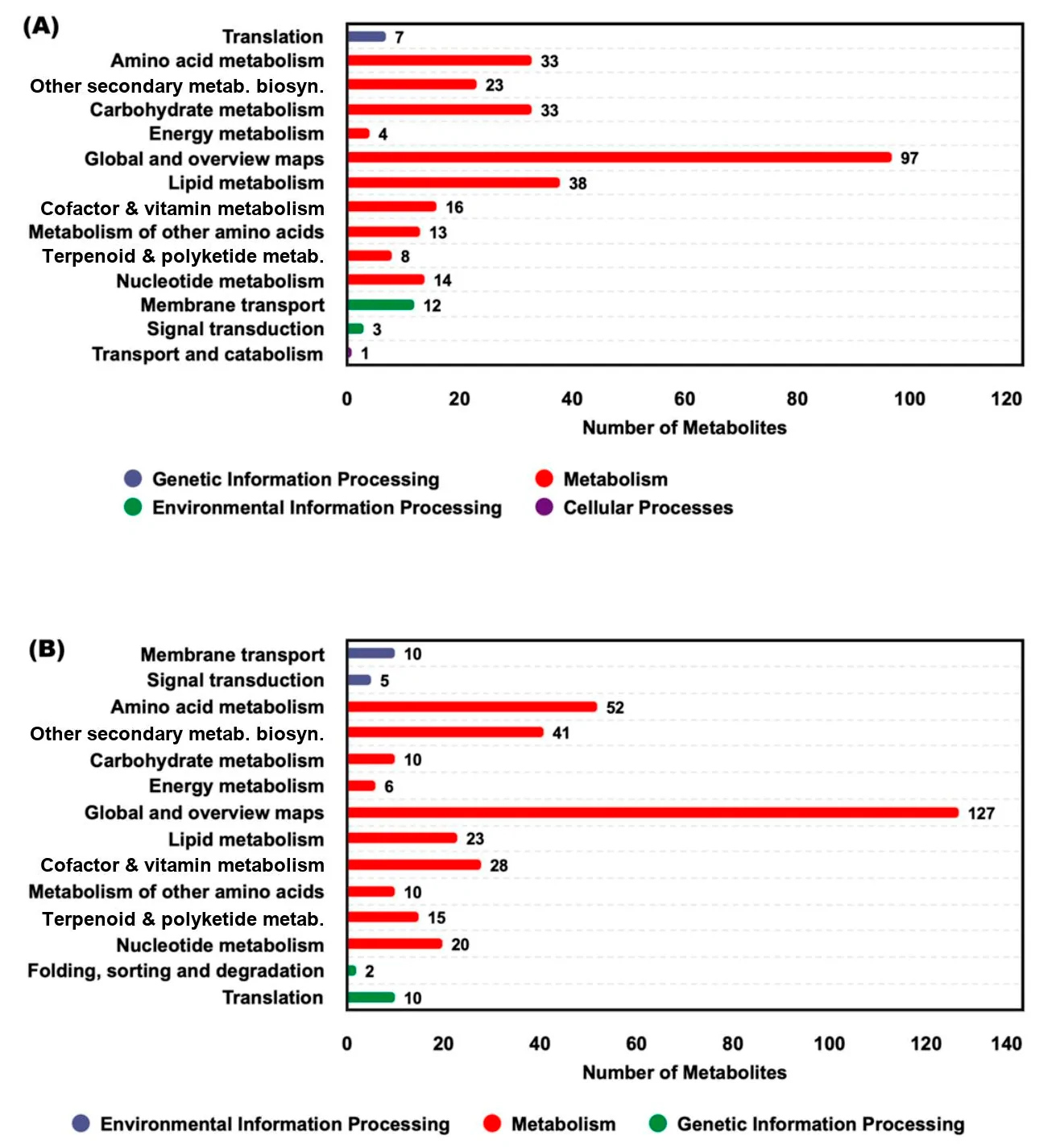
KEGG pathway classification of oat leaf metabolites under low-nitrogen stress. (**A**) KEGG pathway classification of metabolites detected in negative-ion mode; (**B**) KEGG pathway classification of metabolites detected in positive-ion mode. The horizontal axis indicates the number of metabolites annotated to each KEGG pathway category, while different bar colors represent different KEGG functional classifications. Numbers at the ends of the bars indicate the corresponding metabolite counts.

Based on KEGG annotations, the identified metabolites were classified into different functional pathways under negative- and positive-ion modes (Figure 7). In the negative-ion mode, the largest number of annotated metabolites was associated with global and overview maps (97), followed by lipid metabolism (38), amino acid metabolism (33), carbohydrate metabolism (33), and the biosynthesis of other secondary metabolites (23) (Figure 7A). In the positive-ion mode, global and overview maps also contained the largest number of annotated metabolites (127), followed by amino acid metabolism (52), the biosynthesis of other secondary metabolites (41), lipid metabolism (23), and nucleotide metabolism (20) (Figure 7B). Both ionization modes detected metabolites associated with amino acid and lipid metabolism, although the numbers of annotated metabolites differed between the two modes. These differences suggest that negative- and positive-ion modes provide complementary information regarding metabolite detection and KEGG pathway coverage. Overall, the KEGG annotation results provided a reference for the subsequent integrated analysis of transcriptomic and metabolomic data.

To identify the variables contributing to the observed separation, PC1 and PC2 loadings were examined separately for the positive- and negative-ion datasets. PC1 and PC2 explained 29.63% and 16.02% of the variance in the positive-ion dataset and 28.35% and 18.28% in the negative-ion dataset, respectively. The features with the largest absolute positive-ion PC1 loadings included Com_85_pos (putatively annotated as PC O-18:3), Com_4195_pos (dehydroeffusol), Com_224_pos (PC O-18:2) and Com_98_pos (L-leucyl-L-alanine hydrate), whereas Com_2673_pos (deoxycytidine), Com_1332_pos (lysine butyrate), Com_273_pos (2′-deoxyadenosine) and Com_686_pos (N-acetylornithine) had the largest absolute PC2 loadings. In the negative-ion dataset, Com_1306_neg (LPC 18:3), Com_1289_neg, Com_2_neg (LPG 18:3) and Com_310_neg (LPG 18:4) were among the strongest PC1 contributors, whereas Com_101_neg (eicosapentaenoic acid), Com_16_neg (tretinoin), Com_2379_neg and Com_503_neg (prostaglandin K1) had the largest absolute PC2 loadings. The complete ranked lists are provided in Table S2, and the corresponding loading plots are shown in Figure S3.

### 3.8. Pathway-Level Integration of Transcriptomic and Metabolomic Changes Under Low Nitrogen

**Figure 8:**
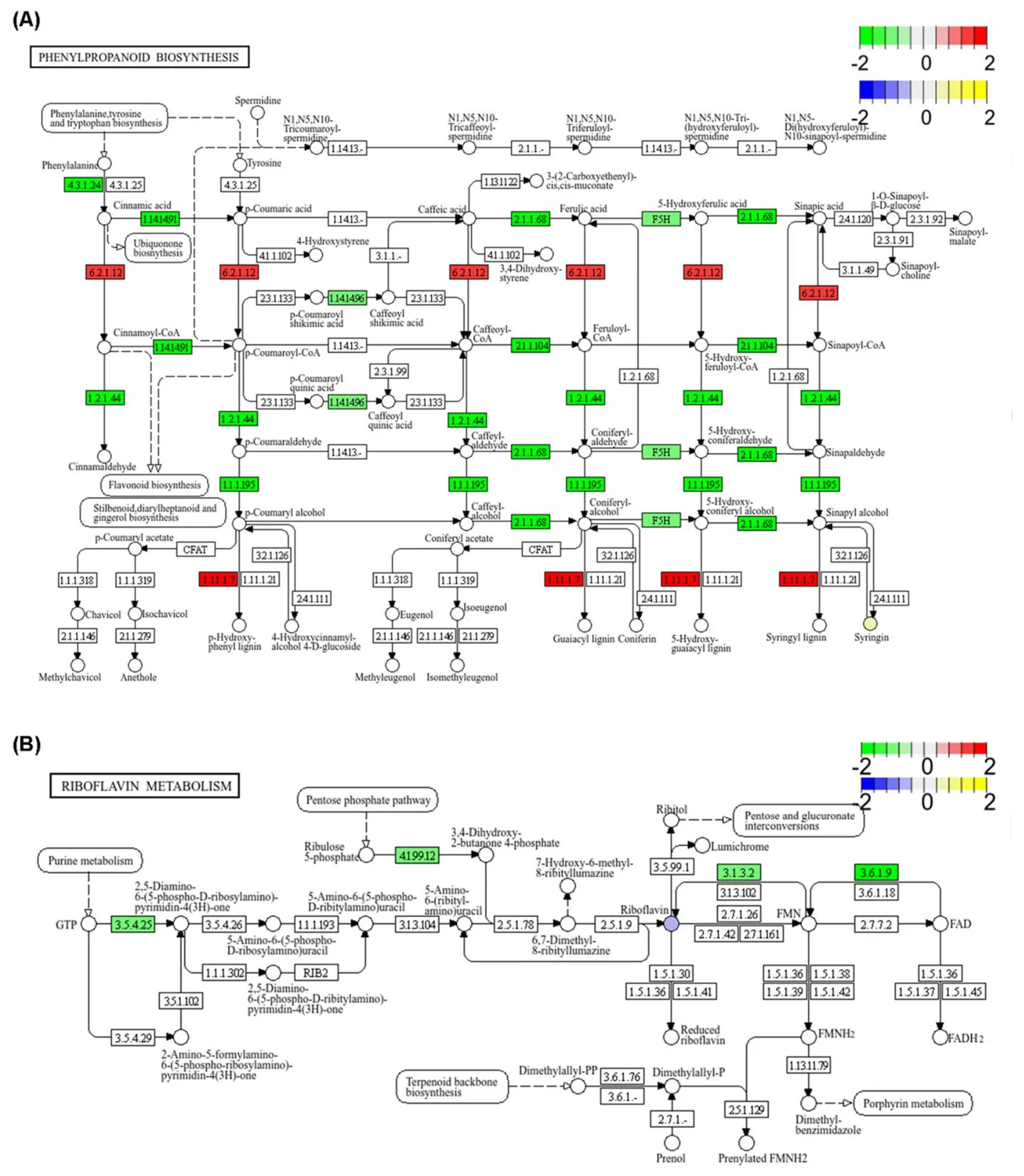
Integrated pathway analysis of differentially expressed genes (DEGs) and differential metabolites (DEMs). (**A**) Phenylpropanoid biosynthesis and (**B**) riboflavin metabolism under low-nitrogen treatment. Rectangles represent enzymes or enzyme-coding genes, while circles represent metabolites. The green-to-red color scale indicates changes in gene expression, whereas the blue-to-yellow color scale indicates changes in metabolite abundance. Arrows indicate the direction of metabolic reactions, and dashed lines indicate connections between metabolic pathways. Numbers within rectangles represent enzyme commission (EC) numbers.

Integrated transcriptomic and metabolomic analysis identified shared pathway annotations, including riboflavin metabolism, phenylpropanoid biosynthesis, and ubiquinone and other terpenoid-quinone biosynthesis (Figure 8). In phenylpropanoid biosynthesis, transcripts annotated as 4-coumarate-CoA ligase (EC 6.2.1.12) and peroxidase (EC 1.11.1.7) increased, whereas transcripts annotated as cinnamoyl-CoA reductase (EC 1.2.1.44), cinnamyl-alcohol dehydrogenase (EC 1.1.1.195), caffeic acid O-methyltransferase (EC 2.1.1.68), and 5-O-(4-coumaroyl)-D-quinate 3′-monooxygenase (EC 1.14.14.96) decreased (Figure 8A). In riboflavin metabolism, transcripts annotated as GTP cyclohydrolase II (EC 3.5.4.25), 3,4-dihydroxy-2-butanone-4-phosphate synthase (EC 4.1.99.12), acid phosphatase (EC 3.1.3.2), and nucleoside diphosphatase (EC 3.6.1.9) also decreased (Figure 8B). These results indicate that low nitrogen was associated with transcript and metabolite changes annotated to phenylpropanoid and riboflavin metabolism.

### 3.9. RNA-Seq Data Validation by qRT-PCR

**Figure 9:**
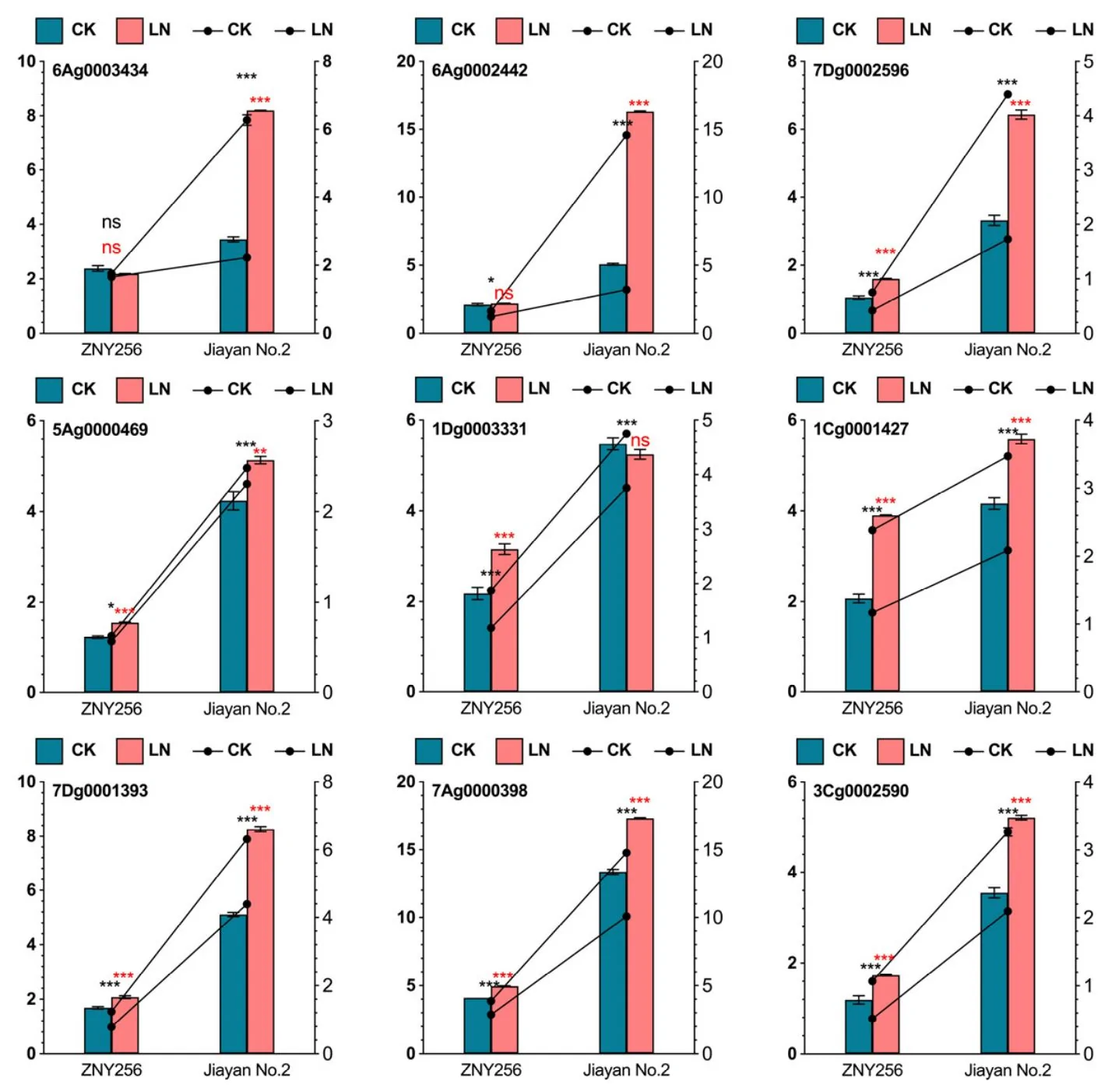
qRT-PCR validation. For the bars with standard errors and broken lines, log2 value of gene changes and log2 fold change were used for qRT-PCR and RNA-seq from three independent biological replicates (*n* = 3) using the 2^−^^∆∆^^CT^ method (left *y*-axis) and the FPKM value of RNA-seq (right *y*-axis), respectively. *, **, and *** indicate *p* < 0.05, *p* < 0.01, and *p* < 0.001, respectively; ns indicates no statistically significant difference (*p* ≥ 0.05).

The expression patterns of nine genes identified from the transcriptome analysis were validated by qRT-PCR in the two oat germplasms under contrasting nitrogen conditions (Figure 9). The selected genes included three genes associated with flavonoid biosynthesis (*AVESA.00001b.r3.6Ag0003434.2*, *AVESA.00001b.r3.6Ag0002442.1* and *AVESA.00001b.r3.7Dg0002596.1*), three genes associated with glutathione metabolism (*AVESA.00001b.r3.5Ag0000469.2*, *AVESA.00001b.r3.1Dg0003331.1* and *AVESA.00001b.r3.1Cg0001427.1*), and three genes associated with phenylpropanoid biosynthesis (*AVESA.00001b.r3.7Dg0001393.2*, *AVESA.00001b.r3.7Ag0000398.1* and *AVESA.00001b.r3.3Cg0002590.10*). All nine genes showed higher expression in the low-nitrogen treatment than in the control in the reported comparison. The direction of expression change measured by qRT-PCR was consistent with the RNA-seq results, supporting the reliability of the transcriptomic expression profiles.

## 4. Discussion

At the 15-day endpoint, low-nitrogen treatment altered glutamine synthetase activity in both oat germplasms, although the magnitude of the response differed between Jiayan No.2 and ZNY256. Changes in nitrate reductase activity, starch phosphorylase activity and soluble sugar content further indicated that nitrogen limitation affected both nitrogen assimilation and carbon metabolism. These differences are consistent with contrasting physiological responses of the two germplasms to low-nitrogen treatment. Under low nitrogen stress conditions, significant germplasm variations in glutamine synthase (GS) activity in oat leaves indicate differences among various oat varieties in terms of nitrogen metabolism adaptability. GGlutamine synthetase (GS) activity was lower under low-nitrogen conditions than under normal-nitrogen conditions, suggesting that oat may adapt to nitrogen limitation by regulating the activity of nitrogen-metabolizing enzymes. In addition, the reduction in the activities of nitrate reductase (NR) and starch phosphorylase (SP), as well as the change in soluble sugar content, further reflect the impact of low-nitrogen stress on the dynamic balance of carbon and nitrogen metabolism in oat. These results suggest that oat may maintain physiological balance by synergistically regulating indicators related to nitrogen assimilation and carbon metabolism. Previous studies have pointed out that low nitrogen stress can lead to a decrease in the activity of nitrogen metabolism enzymes in plants, thereby affecting plant growth and development [17]. Meanwhile, the interaction between nitrogen and carbon metabolism is also an important mechanism for plants to adapt to environmental stress [18].

Under low-nitrogen stress, plants optimize nitrogen utilization efficiency by regulating the expression of key genes. Studies have shown that a low-nitrogen environment can induce the upregulation of genes related to nitrogen metabolism, root development and signal transduction, thereby enhancing the adaptability of plants [19]. In addition, low-nitrogen stress can also trigger the activation of a series of transcription factors, regulating the expression of genes related to nitrogen assimilation and absorption. This process shows significant differences among different plant species [20]. Under low-nitrogen treatment conditions, the up-regulated and down-regulated numbers of differentially expressed genes (DEGs) of Jiayan No.2 and ZNY256 respectively revealed the significant impact of low-nitrogen stress on gene expression, especially the gene expression change of ZNY256 was more obvious, which might be closely related to its role in nitrogen metabolism adaptation. Previous studies have shown that the gene expression patterns of plants undergo significant changes in low-nitrogen environments, especially in genes related to nitrogen assimilation, transport and storage [21]. These studies highlight the strategies by which plants adapt to environmental changes by regulating gene expression under adverse conditions.

Under abiotic stress, the metabolic pathways of plants undergo significant changes. The related KEGG enrichment analysis can reveal the metabolic mechanisms by which plants respond to adverse conditions [22]. Studies have shown that under low-nitrogen conditions, plants enhance their nitrogen assimilation capacity by regulating the KEGG pathway related to nitrogen metabolism, thereby improving their adaptability to growth and development [23]. In addition, GO analysis is often used to identify key genes in specific biological processes. Research has found that plants optimize their nitrogen use efficiency by regulating GO enrichment analysis related to root development and nutrient absorption under low nitrogen stress [24]. Meanwhile, under low-nitrogen stress, plants usually enhance nitrogen utilization efficiency by regulating genes related to stress response and metabolism [25]. In this study, the DEGs of ZNY256 were significantly enriched in the “ribosome biosynthesis” pathway, which is consistent with the increased demand for protein synthesis in plants in response to nutrient deficiencies. In contrast, Jiayan No.2 showed significant enrichment in the carbon fixation pathway of photosynthetic organisms, suggesting that it may adapt to adverse conditions by enhancing photosynthetic efficiency under low-nitrogen conditions. This is consistent with the research results that plants enhance photosynthesis under low-nitrogen conditions. In this study, the DEGs of ZNY256 were significantly enriched in the “ribosome biosynthesis” pathway, which is consistent with the increased demand for protein synthesis in plants in response to nutrient deficiencies [26]. KEGG enrichment analysis revealed different response patterns between the two oat germplasms. In ZNY256, six pathways reached the adjusted significance threshold (*p*adj < 0.05). Because these pathways contained both up- and downregulated DEGs, they are interpreted as reflecting transcriptional reorganization rather than uniform pathway activation. In contrast, no KEGG pathway reached the adjusted significance threshold in Jiayan No. 2. Carbon fixation in photosynthetic organisms, glyoxylate and dicarboxylate metabolism, glutathione metabolism, and photosynthesis were among the highest-ranked pathways, but these trends were not significant after multiple-testing correction. Therefore, they should be regarded as potential response patterns requiring further validation rather than evidence of enhanced pathway activity. In particular, the prominence of carbon fixation- and photosynthesis-related terms alone does not demonstrate enhanced photosynthetic activity under low nitrogen; such an interpretation would require consideration of gene-expression direction together with supporting physiological measurements. This is consistent with the research results that plants enhance photosynthesis under low-nitrogen conditions [27].

In plant research, WGCNA has been widely applied to identify key genes and functional modules related to abiotic stress, revealing co-expression relationships and regulatory networks among different genomes [28]. Studies using WGCNA analysis have found that certain specific gene modules are significantly associated with nitrogen metabolism and stress resistance in plants, providing potential gene targets for improving the growth performance of crops under low-nitrogen conditions [29]. Studies have shown that plants often optimize resource utilization in low-nitrogen environments by adjusting the expression of genes related to photosynthesis and nitrogen metabolism [30]. Based on the cluster analysis results of this study, multiple functional gene modules were identified, especially those related to photosynthesis and nitrogen metabolism, showing significant positive correlations. This indicates that oat may enhance nitrogen utilization efficiency and adaptability under low-nitrogen conditions by coordinating and regulating the genes in these modules. Furthermore, the negative correlation among the modules, especially the association between the amino sugar and nucleotide sugar metabolism modules and the phenylpropanin biosynthesis module, reveals the potential inhibitory or regulatory mechanisms of secondary metabolic pathways under low-nitrogen conditions. The research results indicate that under low nitrogen stress, oat may, through metabolic redistribution, prioritize meeting the demands of nitrogen metabolism and photosynthesis. This strategy may be an important adaptive mechanism for plants to cope with nutritional limitations.

In the research on plants’ response to low nitrogen stress, the regulatory mechanisms of phenylpropanoid biosynthesis and riboflavin metabolism pathways have received considerable attention [31]. Studies have shown that when plants are deficient in nitrogen, they often maintain their physiological balance by regulating metabolic pathways. For instance, they enhance their antioxidant capacity to cope with oxidative stress [32]. The results of this study show that under low nitrogen conditions, genes related to phenylpropanoid biosynthesis, such as 4-coumaric acid—CoA ligase and peroxidase, are significantly upregulated, indicating that plants may respond to adverse environmental stresses by enhancing their antioxidant capacity and cell wall strengthening. However, it is worth noting that the synthesis genes of certain phenylpropanoid metabolites, such as coumaroyl-CoA reductase, linalool dehydrogenase, caffeic acid O-methyltransferase and 5-O-(4-coumaroyl)-D-kynurenic acid 3′-monooxygenase, were significantly downregulated. This may reflect the resource optimization strategy of plants in low-nitrogen environments. There is evidence suggesting that plants often need to reallocate biosynthetic resources in adverse conditions to prioritize the fulfillment of critical physiological processes necessary for survival [33]. Therefore, our results support this view, indicating that the oat plants prioritize the concentration of resources towards nitrogen absorption and utilization under low nitrogen conditions, while reducing the synthesis of certain metabolites. Meanwhile, in the riboflavin metabolism pathway, the genes of GTP cyclase II, 3,4-dihydroxy-2-butanone-4-phosphate synthase, acid phosphatase and nucleoside diphosphatase were significantly downregulated. This further indicates that under low nitrogen conditions, plants have a reduced demand for riboflavin synthesis. This is consistent with previous studies [34], suggesting that plants will reduce the activities of more non-critical metabolic pathways to ensure the prioritized allocation of energy and raw materials when nitrogen is insufficient. The transcript changes and putative metabolite annotations suggest phenylpropanoid biosynthesis and riboflavin metabolism as candidates for further investigation. However, neither pathway mapping nor changes in transcript abundance establish altered metabolic flux, resource allocation, or reduced demand for riboflavin. These interpretations remain hypotheses requiring targeted metabolite measurements and functional experiments.

Jiayan No. 2 maintained growth and nitrogen accumulation better than ZNY256 under the present treatment, providing a phenotypic basis for comparing their contrasting responses. However, substantial transcriptional differences were also present between the two germplasms under control conditions, indicating that genetic background contributed considerably to the observed expression differences. Accordingly, genes, metabolites, and pathways are considered tolerance-associated candidates only when their low-nitrogen responses differ between the two germplasms and are consistent with the better maintenance of performance in Jiayan No. 2. Validation using additional germplasms and functional experiments will be required to establish their causal roles in low-nitrogen tolerance. Previous studies have shown that the synthesis of flavonoids and glutathione is closely related to the responses of plants to environmental stress. Flavonoids can alleviate the oxidative stress that plants endure under low nitrogen conditions due to their antioxidant properties [35]. At the same time, glutathione, as a key antioxidant, can effectively help plants resist stress and maintain the redox balance of cells [36]. Moreover, the biosynthesis of styrene acrylic pigments is closely related to the accumulation of plant pigments and stress resistance [37]. Therefore, the significant high expression of these three types of genes in the experimental group may enhance the adaptability of oat in low nitrogen environments. This study used qRT-PCR technology to verify the expression patterns of 9 genes related to nitrogen tolerance in two oat varieties under low nitrogen stress. The results showed that the gene expression levels in the experimental group were significantly higher than those in the control group, especially for genes related to flavonoid biosynthesis, glutathione metabolism, and styrene acrylic pigment biosynthesis. This finding suggests that these genes may play an important role in the nitrogen tolerance of oat. The candidate genes and metabolites identified in this study should primarily be interpreted as molecular features associated with the response to nitrogen deficiency. Their biological relevance is supported by their functional annotations and their association with metabolic processes altered under low-nitrogen conditions, including nitrogen metabolism, phenylpropanoid and flavonoid biosynthesis, glutathione metabolism, and related antioxidant responses. These processes may reflect adjustments in nitrogen assimilation, carbon allocation, secondary metabolism, and cellular redox homeostasis during nitrogen limitation. However, the present bioinformatics and multi-omics analyses are correlative and do not establish direct regulatory or causal relationships. Therefore, the identified genes and metabolites are considered candidates for further investigation, and their specific roles in the contrasting low-nitrogen responses of the two oat germplasms require functional validation.

## 5. Conclusions

This study characterized responses to low nitrogen in two oat germplasms using phenotypic, physiological, transcriptomic, and exploratory metabolomic observations. Transcriptomic results identified candidate genes associated with low-nitrogen responses. Putative metabolite annotations provided an exploratory context for examining pathways including phenylpropanoid biosynthesis. These findings associate carbon-nitrogen coordination, secondary metabolism, and antioxidant-related processes with contrasting low-nitrogen responses in oat and provide candidates for subsequent functional validation and germplasm evaluation.

## Figures and Tables

**Table 1:** Statistics of the sequencing data quality.

Sample	Raw_Reads (bp)	Raw_Bases (Gb)	Clean_Reads (bp)	Clean_Bases (Gb)	Clean Reads Q30 (%)	GC_Pct (%)
ZNY256_CK_1	43,677,896	6.55	42,400,766	6.36	93.28	54.23
ZNY256_CK_2	39,898,886	5.98	38,406,112	5.76	93.57	54.32
ZNY256_CK_3	43,924,214	6.59	42,419,828	6.36	93.80	53.69
ZNY256_LN_1	45,266,022	6.79	43,786,848	6.57	93.50	54.30
ZNY256_LN_2	42,734,054	6.41	41,735,008	6.26	93.39	54.17
ZNY256_LN_3	41,312,392	6.20	40,222,094	6.03	93.27	53.95
Jiayan No.2_CK_1	42,829,838	6.42	42,035,946	6.31	93.17	52.13
Jiayan No.2_CK_2	41,454,268	6.22	40,271,294	6.04	94.18	53.37
Jiayan No.2_CK_3	45,274,224	6.79	44,118,776	6.62	93.92	54.54
Jiayan No.2_LN_1	41,620,220	6.24	40,430,168	6.06	93.51	53.71
Jiayan No.2_LN_2	48,360,830	7.25	46,672,864	7.00	93.86	51.87
Jiayan No.2_LN_3	47,585,060	7.14	45,989,628	6.90	94.10	53.89

**Table 2:** Statistics of the number of differential genes.

Comparison Group	Up	Down	All
ZNY256_LN vs. ZNY256_CK	2438	1922	4360
Jiayan No.2_LN vs. Jiayan No.2_CK	1050	2129	3179
ZNY256_LN vs. Jiayan No.2_LN	6049	8510	14,559
ZNY256_CK vs. Jiayan No.2_Ck	7204	10,919	18,123

## Data Availability

All data generated or analyzed during this study are included in this article. Additional raw data are available from the corresponding author on reasonable request.

## Supplementary Materials

The following supporting information can be downloaded at https://www.mdpi.com/article/10.3390/cells15201874/s1, Figure S1: Gene Ontology (GO) enrichment analysis of differentially expressed genes (DEGs) in oat leaves under low-nitrogen stress; Figure S2: Kyoto Encyclopedia of Genes and Genomes (KEGG) pathway enrichment analysis of differentially expressed genes (DEGs) in oat leaves under low-nitrogen stress; Figure S3: Principal component analysis (PCA) loading plots of metabolite features detected in oat leaves in positive- and negative-ion modes; Table S1: Primer sequences used for quantitative real-time PCR (qRT-PCR) analysis of selected genes in oat (*Avena sativa* L.); Table S2: Top 20 metabolite features ranked by absolute principal component analysis (PCA) loading values for PC1 and PC2 in positive- and negative-ion modes.

## Abbreviations

The following abbreviations are used in this manuscript:

ABC	ATP-binding cassette
CK	Normal-nitrogen control
CV	Coefficient of variation
DEG	Differentially expressed gene
FC	Fold change
FPKM	Fragments per kilobase of transcript per million mapped reads
GO	Gene Ontology
GS	Glutamine synthetase
GTP	Guanosine triphosphate
HMDB	Human Metabolome Database
KEGG	Kyoto Encyclopedia of Genes and Genomes
LC–MS/MS	Liquid chromatography–tandem mass spectrometry
LN	Low nitrogen
MAPK	Mitogen-activated protein kinase
MS/MS	Tandem mass spectrometry
*m*/*z*	Mass-to-charge ratio
NR	Nitrate reductase
OD260/280	Ratio of absorbance at 260 nm to 280 nm
PCA	Principal component analysis
PLS-DA	Partial least squares discriminant analysis
QC	Quality control
qRT–PCR	Quantitative reverse-transcription polymerase chain reaction
RIN	RNA integrity number
RNA-seq	RNA sequencing
SP	Starch phosphorylase
VIP	Variable importance in projection
WGCNA	Weighted gene co-expression network analysis
