# Supplementary material for: Integrated Transcriptomic and Metabolomic Analyses Reveal Leaf Regulatory Mechanisms Underlying Low-Nitrogen Tolerance in Oat (Avena sativa L.)
Source: Cells. 2026 Oct 15;15(20):1874. doi: 10.3390/cells15201874 (PMC13703495; doi:10.3390/cells15201874)
Supplement: Supplementary file 1 [file cells-15-01874-s001.zip › Supplementary_Captions.pdf]

Supplementary Figure Legends and Table Titles

Figure S1. Gene Ontology (GO) enrichment analysis of differentially expressed genes (DEGs) in oat leaves under low-nitrogen stress. (A) Jiayan No. 2, low nitrogen (LN) versus control (CK); (B) ZNY256, LN versus CK. The horizontal axis shows GeneRatio, and the vertical axis shows GO terms. Bubble size represents the number of DEGs associated with each term (Count), and bubble color indicates the adjusted p-value (padj), with red denoting lower adjusted p-values.

Figure S2. Kyoto Encyclopedia of Genes and Genomes (KEGG) pathway enrichment analysis of differentially expressed genes (DEGs) in oat leaves under low-nitrogen stress. (A) Jiayan No. 2, low nitrogen (LN) versus control (CK); (B) ZNY256, LN versus CK. The horizontal axis shows GeneRatio, and the vertical axis lists KEGG pathways. Bubble size represents the number of DEGs associated with each pathway (Count), and bubble color indicates the adjusted p-value (padj), with red denoting lower adjusted p-values.

Figure S3. Principal component analysis (PCA) loading plots of metabolite features detected in oat leaves in positive- and negative-ion modes. (a) Twenty metabolite features with the largest absolute loadings on PC1 in positive-ion mode (29.63% of variance); (b) PC2 in positive-ion mode (16.02%); (c) PC1 in negative-ion mode (28.35%); and (d) PC2 in negative-ion mode (18.28%). The horizontal axis represents PCA loadings and the vertical axis lists metabolite features. Blue-green bars indicate positive loadings, while orange-red bars indicate negative loadings. Loading magnitude indicates the strength of association with the corresponding principal component.

Table S1. Primer sequences used for quantitative real-time PCR (qRT-PCR) analysis of selected genes in oat (*Avena sativa* L.). Gene identifiers and corresponding forward and reverse primer sequences are provided; primer sequences are shown in the 5′–3′ direction.

Table S2. Top 20 metabolite features ranked by absolute principal component analysis (PCA) loading values for PC1 and PC2 in positive- and negative-ion modes. Information includes ionization mode, principal component, rank, feature identifier, metabolite annotation, molecular formula, molecular weight, retention time (RT), mass-to-charge ratio (m/z), metabolite classification, quality-control coefficient of variation (QC-CV), loading value, and absolute loading value.

Note for final verification: Panels A and B of Figures S1 and S2 are assigned to Jiayan No. 2 (LN vs. CK) and ZNY256 (LN vs. CK), respectively, as confirmed. Check that this order matches the source plotting files before submission.
